# Assessment of Diagnostic Value of Post Mortem Tongue Tip Fluids for Disease Detection in Growing Pigs

**DOI:** 10.3390/ani15162434

**Published:** 2025-08-19

**Authors:** Claudio Marcello Melini, Mariana Kikuti, Xiaomei Yue, Igor A. D. Paploski, Albert Canturri, Stephanie Rossow, Brad Leuwerke, Steve Stone, Cesar A. Corzo

**Affiliations:** 1 Department of Veterinary Population Medicine, University of Minnesota, Saint Paul, MN 55108, USA; melin145@umn.edu (C.M.M.); mkikuti@umn.edu (M.K.); yue00075@umn.edu (X.Y.); ipaplosk@umn.edu (I.A.D.P.); albert.canturri@regeneron.com (A.C.); 2 Veterinary Diagnostic Laboratory, University of Minnesota, Saint Paul, MN 55108, USA; 3 Swine Vet Center, Saint Peter, MN 56082, USA; bleuwerke@swinevetcenter.com; 4 Fairmont Veterinary Clinic, Fairmont, MN 56031, USA; sstone@fmtvets.com

**Keywords:** post mortem, tongue tip fluid, growing pigs, alternative specimens

## Abstract

Assessing whether post mortem samples can provide value for monitoring during the post-weaning stages is necessary as this methodology can be adopted by the industry when resources are scarce. The objectives of this study are to assess pathogen detection in tongue tip fluids (TTFs), oronasal swabs (ONSs), rectal swabs (RSs), and superficial inguinal lymph nodes (SILNs). Two farms (one wean-to-finish farm and one finishing farm) undergoing a porcine reproductive and respiratory syndrome virus (PRRSV) outbreak were included, and 30 dead pigs were sampled during each farm visit. For PRRSV, the diagnostic accuracy of TTF, ONS, RS, and SILN were calculated using intracardiac serum as the gold standard. We also characterized the detection of PCV-2, PCV-3, PPV-1, PPV-2, *Lawsonia intracellularis*, and IAV in all collected specimens. Overall, TTF and SILN had the highest sensitivity for PRRSV-2 detection (95% and 99%, respectively). Most other pathogens were detected in TTF, with the highest detection rates were for PPV-2 (99%) and PCV-2 (41%). RS had mostly good detection rates for PPV-2 (27–73%), while ONS had similar detection rates (37%) to TTF (38%) for IAV. Altogether, this indicates that post mortem specimens can provide value for pathogen detection.

## Simple Summary

Traditional collected samples often require skilled staff or additional working time, which can be limited by farm resources. We aimed to explore whether easily collected samples from dead pigs can be used to detect important swine pathogens in growing pigs. We collected blood directly from the hearts of dead pigs and compared it to other samples for the presence of porcine reproductive and respiratory syndrome, and tested different samples for several common pig viruses and bacteria. Results showed that tongue tip samples could be used to detect six out of the seven pathogens being studied. While tongue tip fluid samples were not perfect for detecting every pathogen, they provided useful information and may be a practical option when more complex sampling is not feasible.

## 1. Introduction

The swine industry continues to develop methodologies for specimen collection that optimizes farm personnel time while providing accurate diagnostic results. Collecting individual live pig samples like blood via jugular venipuncture is a time-consuming activity and demands a certain skill level, and, as such, most post-weaning live sampling relies on oral fluid. Post mortem tissue sampling, meanwhile, depends on whether farm personnel possess the necessary skills to conduct a proper post mortem examination and sample collection [1]. As an alternative, easy-to-collect post mortem specimens, such as stillbirths, placenta, processing fluids, or the recently reported tongue tip fluid (TTF), have been proposed for porcine reproductive and respiratory syndrome (PRRS) monitoring in breeding herds, particularly when processing fluid collection is not feasible [2,3,4,5]. However, information on the use of TTF in nursery and finisher pigs in the United States is scarce. Therefore, it is necessary to assess whether post mortem specimens including TTF, intracardiac blood (ICB), oronasal swabs (ONSs), rectal swabs (RSs), and superficial inguinal lymph nodes (SILNs) can provide a valuable alternative for screening and monitoring in the post-weaning stages. Although these specimens may not be the gold standard for detecting certain swine pathogens, they can still provide value if adopted by the industry given current labor availability challenges.

Even though the collection of post mortem samples requiring a full necropsy is still a necessary procedure when investigating sudden morbidity and mortality increase, this process requires time and a specific set of skills from field personnel, which makes TTF a rapid alternative that can precede full post mortem examination. Therefore, TTF is an animal-welfare friendly post mortem specimen that provides practitioners and producers with a powerful screening tool that can avoid causing major environmental contamination.

To assess if TTF, ICB, ONS, RS, and SILN can be alternative specimens for specific pathogen screening in growing pigs’ mortality, we designed a study to address the following objectives: (1) to assess the sensitivity, specificity, and positive and negative predictive values of TTF, ONS, RS, and SILN in growing pigs when compared to ICB in populations of pigs undergoing a PRRSV-2 outbreak; and (2) to describe the detection frequency of commonly found growing-pig pathogens (e.g., porcine circovirus type 2 [PCV-2], porcine circovirus type 3 [PCV-3], porcine parvovirus type 1 [PPV-1], porcine parvovirus type 2 [PPV-2], influenza A virus [IAV]) and *Lawsonia intracellularis* through reverse transcription-PCR (RT-PCR)/PCR in TTF and target specimens.

## 2. Materials and Methods

### 2.1. Study Design

One 2400-head wean-to-finish farm (Farm 1) and one 3300-head grow-finish farm (Farm 2) located in the state of Minnesota were conveniently selected for this exploratory study. The farms were selected based on the PRRS status of both the growing pigs and their source sow herd. Specifically, the eligible farm should have had an ongoing PRRSV-2 wild-type outbreak or be housing placed positive pigs. However, at the time of the collection, the source breeding farm was required to have been classified as category 3 or 4 according to the American Association of Swine Veterinarians (AASV) PRRS guidelines [6]. The selected growing-pig population should also have been unvaccinated for PRRSV-2, but Farm 1 pigs were vaccinated for PCV-2 + *Mycoplasma hyopneumoniae* (Fostera^®^ Gold PCV-MH, Zoetis, Parsippany, NJ, USA), *L. intracellularis* (Porcilis^®^ Ileitis, MERCK Animal Health, Rahway, NJ, USA), and *Erisipelothrix rhusiopathiae* (RespiSure-ONE^®^/ER Bac Plus^®^, Zoetis, Parsippany, NJ, USA) at 22 days of age (weaning) and three weeks post weaning. Farm 2 pigs were vaccinated for PCV-2 (Fostera^®^ Gold PCV, Zoetis, Parsippany, NJ, USA) at 21 days of age (weaning), and *Salmonella cholerasuis*/*typhimurium* (Argus^®^ SC/ST, MERCK Animal Health, Rahway, NJ, USA) and *Escherichia coli* (Edema Vac^®^, ARKO Laboratories, Jewell, IA, USA) 7 to 14 days post weaning. Farm 1 was visited twice: at 6 (Visit 1—three-week post-placement) and 12 (Visit 2—nine weeks post-placement) weeks of age (WOA). Farm 2 was visited once at 15 WOA (Visit 3—five weeks post-placement).

During each farm visit, a total of 30 dead pigs were selected for sample collection. This sample size enabled researchers to be 95% confident of detecting at least 1 PRRSV-2 PCR-positive pig when the within-herd prevalence was at least 10%, and was calculated using R software version 4.4.0 [7]. Therefore, a total of 90 dead pigs were used for sample collection.

At least one day prior to the farm visit, the veterinarian and farm personnel were informed of our visit and asked to place dead or recently euthanized pigs in a specific area of the barn for researchers to collect samples. Pigs included in this study were those that died naturally of unknown causes or those that required humane euthanasia, a process that was conducted following the American Veterinary Medical Association (AVMA) guidelines for the euthanasia of animals [8].

### 2.2. Specimen Collection and Processing

A systematic process was developed to collect specimens with the goal of maintaining sample identification integrity and avoiding cross contamination. Researchers wore gloves throughout the entire sampling process. Gloves were changed and hands were cleaned with disinfectant wipes between each pig and each specimen. Briefly, (1) ICB was collected while the pig was placed in lateral recumbency, and a sterile syringe and a 16G × 3″ needle were inserted into the thoracic cavity into the heart, with 3–5 mL of blood being retrieved and then placed into a sterile blood collection tube (BD Vacutainer^®^, Becton, Dickinson and Company, Franklin Lakes, NJ, USA); (2) the ONS sample was collected by inserting the swab (BBL™CultureSwab™, Copan Italia S.p.A., Brescia, Italy) into the nasal cavity and then the oral cavity using rotating movements; (3) the RS was collected by inserting the swab into the rectum; (4) the tongue tip (TT) was collected using a new and disposable scalpel and clean forceps. A 1-inch section of the tip of the tongue was removed and placed into a sterile bag (Whirl-pak^®^, MilliporeSigma, Burlington, MA, USA); (5) one SILN was obtained through dissection using a new disposable scalpel and decontaminated forceps and placed into a sterile bag.

Samples were consecutively labeled individually and refrigerated while in-transit to the University of Minnesota Veterinary Diagnostic Laboratory (UMN-VDL). In preparation for submission, TT samples were stored at −20 °C for 12 h and thawed. Upon thawing, TTs were processed individually following the methodology of [2,3], but adapted to accommodate the testing of five pathogens. A total of 2000 µL of phosphate-buffered saline solution (PBS) was added to each bag and manually homogenized for one minute until the fluid mixed with the thawed tongue tip fluid (TTF). Two 1000 µL TTF aliquots were obtained. Each ONS and RS was placed in 1800 µL of Dubelcco’s modified eagle medium (DMEM) (Gibco, ThermoFisher Scientific, Waltham, MA, USA) and vortexed for 15 s, obtaining two 900 µL aliquots. The ICB samples were centrifuged at 1500× *g* for 10 min at 4 °C to obtain two 1000 µL aliquots of intracardiac serum (ICS). Samples, including unprocessed SILN, were then submitted to the UMN-VDL for testing.

### 2.3. Diagnostic Testing

High-throughput total nucleic acid extraction was performed using magnetic bead technology (MagMAX Core Nucleic Acid Purification Kit, ThermoFisher Scientific, Waltham, MA, USA). Samples were individually tested for PRRSV-1 and PRRSV-2 by RT-PCR ThermoFisher VetMAX™ PRRSV EU and NA 3.0 Kit (ThermoFisher Scientific, Waltham, MA, USA) and the National Animal Health Laboratory Network Influenza-A Matrix Assay protocol for IAV, respectively. PCR, Indical Bioscience Virotype PCV-2, and PCV-3 Primers and Probes, and Indical Bioscience Virotype +IC (JOE)-DNA, were used for PCV-2 and PCV-3, while PCR targeting VP2 for PPV-1 and PPV-2 [9], and an in-house PCR assay designed to detect *L. intracellularis* [10], were used.

The TTFs were tested for PRRSV-2, PCV-2, PCV-3, PPV-1, PPV-2, IAV, and *L. intracellularis*. The ONSs were tested for both PRRSV-2 and IAV. The RS samples were tested for PPV-1, PPV-2, and *L. intracellularis*. ICS was tested for PRRSV-2, and SILNs were tested for PRRSV-2, PCV-2, and PCV-3. As per UMN-VDL interpretation, results were considered positive for PCV-2, PCV-3, PPV-1, PPV-2, IAV, and *L. intracellularis* if the cycle threshold (Ct) values were below 36.0. For PRRSV-2, results were considered positive if the Ct values were below 40.0. Two samples of TTF, one with the lowest Ct value and the other equal to 30, which is a high value that could be considered for sequencing if there are no lower alternatives, were submitted for each of the seven pathogens for sequencing.

### 2.4. Data Analysis

Results for PRRSV-1 were not included in the analysis as this PRRSV species is not commonly found in the U.S. and negative results were expected. The detection of PRRSV-2 in TTF, ONSs, and SILNs was compared through the calculation of sensitivity (Se), specificity (Sp), positive predictive value (Ppv), and negative predictive value (Npv) [3,11], using ICS as the gold standard specimen. For the assessment of the detection of PCV-2, PCV-3, PPV-1, PPV-2, IAV, and *L. intracellularis* through RT-PCR and PCR using TTF, ONSs, RSs and SILN specimens, none are to be considered as a substitute for their respective gold-standard specimens and diagnostic tools. Results were compared descriptively based on the proportion of samples in which each pathogen was detected by the specimen. And for all pathogens and their collected specimen’s detection, agreement was calculated using Cohen’s kappa and overall agreement. Analysis was performed using the base, psych [12], and vcd [13] packages of R software [7].

## 3. Results

The sample size of 30 dead pigs was met for both visits to Farm 1 and for the one visit to Farm 2. The number of pigs that were euthanized by farm personnel was 15 out of 30 for Visit 1, 26 out of 30 for Visit 2, and 25 out of 30 for Visit 3. All samples were successfully collected and tested for PRRS RT-PCR except from three ICB samples collected during the Visit 1, as they lacked sufficient volume.

### 3.1. Pathogen Detection in Post Mortem Specimens

All pathogens, except for PCV-3, were detected at least once in TTF. The proportion of positive samples varied by age and specimen, with PRRSV-2 and PPV-2 being detected more frequently, while PPV-1 and *L. intracellularis* were rarely detected (Table 1). There was a high detection level of PRRSV-2 in TTF as 95% (86/90) of all the collected samples tested positive. The detection of PRRSV-2 in other specimens followed a similar trend to TTF, with the virus being detected in all but one SILN and in approximately 86% (77/90) of the ONS samples. The lowest median Ct values were identified in SILNs followed by serum.

**Figure 1:**
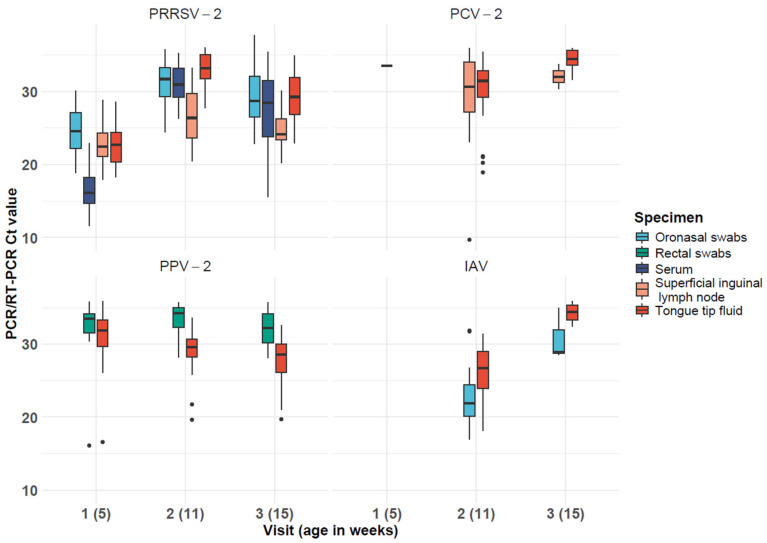
Results in Ct values from PCR/RT-PCR for PRRSV-2, PCV-2, PPV-2, and IAV by visit and specimen.

In general, the Ct values for PRRSV-2 ranged from 18.9 to 37.8 in ONSs, 17.9 to 33.3 in SILNs, 11.6 to 35.5 in serum, and 18.3 to 36.0 in TTF (Table 1 and Figure 1). These values appear to be lower at Visit 1, especially in serum. As for the rest of the pathogens, we only considered those with a Ct value below 36.0, and the results are as follows. For PCV-2, the Ct values ranged from 9.7 to 35.9 in SILNs, and from 18.9 to 35.9 in TTF, with lower TTF values mostly detected during Visit 2. For PPV-1, only one detection in TTF was found during Visit 3 with a Ct value of 25.5. For PPV-2, Ct values ranged from 16.1 to 35.8 in RSs and from 16.6 to 35.9 in TTF, with the latter being apparently lower during all three visits. For *L. intracellularis*, results were obtained from Visit 3 in TTF, with Ct values from 34.3 to 35.5. Lastly, for IAV Ct, values ranged from 17.0 to 35.0 in ONS and from 18.1 to 35.9 in TTF, mostly from samples collected during Visit 2. Tongue tip fluid sequencing results were obtained from one out of two samples for PRRSV-2 using ORF5, from two out of two samples for PCV-2, from zero out of two samples for PPV-2 VP2 using Sanger sequencing, from two out of two samples for IAV using H gene full-length sequencing, and from zero out of one sample for *L. intracellularis*.

Overall, TTF led to a higher detection of PCV-2, PPV-1, PPV-2, IAV, and *L. intracelullaris* when compared to other specimens. However, PCV-3 was not detected in either TTF or SILNs in any of the visits.

### 3.2. Sensitivity, Specificity, and Predictive Values of PRRSV-2 Post Mortem Specimens

From Visits 1 and 3, PRRSV-2 was detected in all TTF, ICS, ONS, and SILN specimens, from which only Se and Ppv could be calculated (Table 2). Visit 2 had a different dynamic as not all specimens had positive results, and from the calculations ONS appear to have lower Se than TTF and SILN but better Sp than those specimens.

### 3.3. Pathogen Detection Agreement Among Post Mortem Specimens

Agreement between two specimens was calculated for each pathogen using Cohen’s kappa (Table 3). For PRRSV-2 the highest agreement was between ICS and ONS and the lowest was between SILN and TTF, but when calculating the global agreement, the best result was between SILN and TTF, and the lowest was between ICS and TTF. With the exception of IAV, the rest of the pathogens showed no level of agreement with Cohen’s kappa, but an acceptable level of global agreement.

## 4. Discussion

Health monitoring in live growing-pig herds currently relies on oral fluid as the main specimen due to its easiness to collect, lack of pig handling involved, and acceptable diagnostic performance; however, alternatives to post mortem sample collection are needed [1,14,15,16,17,18,19,20,21,22,23]. Our study confirmed that TTF can be a practical post mortem specimen that the industry can use when screening for PRRSV; our findings agree with what has been recently reported regarding the value of this specimen in piglets by Baliellas [2], Kikuti [3], and Machado [5], and in growing pigs by Baliellas [2] and Osemeke [24]. Moreover, it can help farm personnel and veterinarians to reduce the collection time using targeted specimens, as recognizing macroscopical lesions from different tissues requires training and time, especially if it has to be performed on several carcasses. Finding PRRSV-2 via RT-PCR in ONSs, SILNs, ICSs, and TTF varied between collection points in Farm 1. Interestingly, between 84% and 99% of the overall samples were positive, with the lowest Ct values found in the ICSs, followed by SILNs, TTF, and ONSs (Table 1).

The detection of other pathogens on TTF besides PRRSV-2 has been reported for IAV [24] and PCV-2 [25]. Our study confirmed that PCV-2, PPV-2, IAV, and *L. intracellularis* can be detected in TTF via molecular diagnostics and, in some cases, the detection of these pathogens had a higher positivity rate in TTF when compared to other specimens. They also had high global agreement, but when calculating Cohen’s kappa there was mostly a lack of agreement, with the exception of IAV. Such differences may be attributed to other reasons besides the pig truly being infected, for example, environmental contamination. It is known that PCV-2 and PPV can be stable in the environment for a prolonged period of time [26,27,28,29]. With regard to PCV-2, we primarily detected this virus in TTF rather than SILN, with a similar range of Ct values between specimens. This virus has been previously detected through PCR/qPCR in nasal swabs, oropharyngeal swabs, serum, and feces [30,31,32,33], suggesting that pigs can shed the virus into the environment, making it ubiquitous, and because of the pigs’ natural rooting behavior, the virus contaminated the oral cavity. Even though PCR detection of PCV-2 on SILN can be more sensitive than in situ hybridization (ISH) [34], failure to detect it in our study could be the result of the pigs being protected by the vaccine and not developing the disease [35]. Although the detection of PCV-2 using PCR on TTF and SILN was possible, the combination of histopathology with immunohistochemistry (IHC) or ISH in tissues remains the primary option for diagnostics [36,37,38]. In the case of PPV, the virus has been detected in several specimens (e.g., serum, lung, heart, liver, kidney, spleen, lymph nodes, duodenum, tonsil, and feces) through PCR/qPCR [39,40,41,42], giving personnel several sampling options. We were only able to detect PPV-1 in one TTF sample, indicating that this virus did not seem to be present at high prevalence in the studied populations; however, PPV-2, a more prevalent virus, was detected with a higher frequency and with similar Ct values between TTF and RS. As with PCV-2, we believe this difference could be in part the result of its ubiquitous nature rather than the population of pigs; however, in Visits 1 and 2, the population of pigs was actively shedding, while in Visit 3 fewer older pigs were shedding, supporting our theory of environmental contamination leading to a high positivity rate of PPV-2 in TTF.

In the case of IAV, it was detected in the same number of ONSs and TTF samples during Visit 2. This result was expected, as both the nasal and oral cavity are linked and nasal secretions can end up in the oral cavity. The nasal swab is the main specimen for IAV RT-qPCR detection in live pigs of different ages [16,43,44,45], but here we provide evidence that TTF can be an alternative when dealing with dead pigs that were shedding and in the absence of appropriate sampling materials, or as a more economic choice. The virus was not detected during Visit 1 or Visit 3, perhaps due to infection dynamics and its low prevalence. However, despite the fact that IAV can be shed for 5–7 days [43,46], and that its RNA can be present on different surface types for hours or days [47,48,49,50], TTF can aid in the detection of the virus at quantities that can yield a sequence or perhaps attempt isolation.

Lastly, *L. intracellularis* has the ability to survive ex vivo in air and feces between three hours to 14 days [51,52]. It is considered widespread in the pig population [53], and can be shed in feces from 2 to 12 weeks post-inoculation [54,55,56], which facilitates its fecal–oral transmission route [55]. Still, in our study it was only detected in 5 out of the 90 TTF samples, but in no RSs. This finding could be the result of a low-prevalence scenario leading to environmental contamination, which was captured by the pigs’ oral cavities. The pigs in this group had been vaccinated against this pathogen and it has been reported that the vaccine does reduce the quantity of the pathogen being shed due to fewer intestinal lesions [57].

Obtaining sequences from TTF is also possible, which provides one more alternative for practitioners conducting diagnostic investigation. We successfully obtained PRRSV-2 sequences from two TTF samples, demonstrating the feasibility of this approach. However, further interpretation of these sequences may be challenging, as tongue tips may also capture viruses from other animals in the herd due to natural chewing behaviors [3]. Despite this limitation, TTs offer the key advantage of enabling testing of a larger number of animals using a sample type that requires minimal labor for collection and that requires no handling and stressing of live animals.

Our study has limitations regarding sample size, within-herd prevalence, and farm follow-up. A small sample size per farm was used in this exploratory study, and generalization to other growing herds may be depend on health management criteria (e.g., vaccination and euthanasia protocols), while the pig sample size was determined based on PRRS, which could have underestimated the power to detect the other pathogens included in this study. Even though the results of this study confirm the detection of several pathogens from TTF, further evidence is needed to better understand the real value of this specimen to differentiate environmental contamination from presence in pigs; we suggest adding environmental sampling to the surfaces of the pens where the mortality originated from and comparing them to the findings of TTF, if the goal is to compare the detection of pathogens. Furthermore, this study focused on PRRSV and enrolled farms experiencing an outbreak which led to a high PRRSV prevalence scenario; however, it is unknown how TTF will perform under mid- or low-prevalence scenarios in growing herds, compared to what has been described in breeding herds [3]. While not a direct comparison, the other pathogens included in this study could have represented potential mid- or lower-prevalence scenarios, depending on the age at sampling. An example of this was IAV in Visit 3, which was conducted when the prevalence seemed to be lower compared to Visit 2, and TTF still contributed to detection. Additionally, inhibitors in fecal samples [58] may have affected the detection of *L. intracellularis* and PPV-1, as it has been mentioned as a possibility for PRRSV-2 detection using fecal swabs [3], but this would not explain why this did not affect the detection of PPV-2. A practical concern of our study is that an important number of pigs were humanely euthanized because of the infectious disease outbreak, which allowed us to meet our required sample size; however, this number of available pigs may not occur often on a single day. Although the goal of this study was not to assess the cost of specimen collection and labor efficiency for TT collection, we believe that TTs may be a cheap and labor-efficient specimen to collect in scenarios in which testing live animals is difficult. However, a formal comparison of the labor and time need to be conducted. Overall, TTF is an easy to collect, welfare-friendly specimen for pathogen detection. Sample collection requires minimal skills and materials, as the oral cavity is easily accessible and sectioning the tip of the tongue requires tools that may be readily available in pig farms.

## 5. Conclusions

Our study provides evidence that confirms that in the growing-pig herd, different swine pathogens could be detected using post mortem specimens, including TTF. Pathogen detection in TTF can vary according to the pathogen, given the infection dynamics of the population in question. Overall, the performance of TTF for PRRSV in growing pigs needs more evaluation to assess if it can be used beyond the detection of pathogens and more as an alternative for health monitoring. Indeed, a complete and exhaustive collection of multiple clinical specimens from different body systems remains the gold standard in diagnostic investigation.

## Figures and Tables

**Table 1:** Proportion of PCR/RT-PCR positive specimens by visit and pathogen according to age in two growing-pig farms undergoing a PRRSV-2 outbreak.

Pathogen	Specimen *	Number of Positive Samples by Pathogen and Ct Values of Positive Samples by Farm and Visit									Overall		
		Farm 1						Farm 2					
		Visit 1 (6 Weeks of Age)			Visit 2 (12 Weeks of Age)			Visit 3 (15 Weeks of Age)					
		n/N (%)	Median (IQR)	Min, Max	n/N (%)	Median (IQR)	Min, Max	n/N (%)	Median (IQR)	Min, Max	n/N (%)	Median (IQR)	Min, Max
PRRSV-2	ONS	30/30	24.5	18.9, 30.1	17/30	31.7	24.5, 35.8	30/30	28.7	22.8, 37.8	77/90	28.1	18.9, 37.8
		(100%)	(22.2, 27.3)		(57%)	(29.3, 33.3)		(100%)	(26.5, 32.1)		(86%)	(24.9, 31.6)	
	SILN	30/30	22.3	17.9, 28.9	29/30	26.4	20.5, 33.3	30/30	24.2	20.2, 30.1	89/90	24.1	17.9, 33.3
		(100%)	(20.9, 24.0)		(97%)	(23.6, 29.7)		(100%)	(23.3, 26.3)		(99%)	(22.1, 26.9)	
	ICS	27/27 ¶	16.1	11.6, 22.9	19/30	31.0	26.3, 35.3	27/30	28.5	15.6, 35.5	73/87	26.3	11.6, 35.5
		(100%)	(14.7, 18.2)		(63%)	(29.3, 33.2)		(90%)	(23.8, 31.5)		(84%)	(17.1, 31.1)	
	TTF	30/30	21.6	18.3, 27.2	26/30	33.2	27.7, 36.0	30/30	29.3	22.9, 34.9	86/90	28.4	18.3, 36.0
		(100%)	(20.0, 23.7)		(87%)	(31.8, 35.0)		(100%)	(26.8, 32.0)		(96%)	(23.6, 32.6)	
PCV-2	SILN	0/30	-	-	8/30	30.6	9.7, 35.9	2/30	32.0	30.4, 33.7	10/90	30.8	9.7, 35.9
		(0%)	-		(27%)	(27.2, 34.0)		(7%)	(31.2, 32.9)		(11%)	(28.9, 33.7)	
	TTF	1/30	33.5	-	27/30	31.5	18.9, 35.4	9/30	34.4	31.7, 35.9	37/90	32.2	18.9, 35.9
		(3%)	(-,-)		(90%)	(29.2, 32.8)		(30%)	(33.6, 35.7)		(41%)	(30.5, 34.4)	
PCV-3	SILN	0/30	-	-	0/30	-	-	0/30	-	-	0/90	-	-
		(0%)	-		(0%)	-		(0%)	-		(0%)	-	
	TTF	0/30	-	-	0/30	-	-	0/30	-	-	0/90	-	-
		(0%)	-		(0%)	-		(0%)	-		(0%)	-	
PPV-1	RS	0/30	-	-	0/30	-	-	0/30	-	-	0/90	-	-
		(0%)	-		(0%)	-		(0%)	-		(0%)	-	
	TTF	0/30	-	-	0/30	-	-	1/30	25.5	-	1/90	25.5	-
		(0%)	-		(0%)	-		(3%)	(-,-)		(1%)	(-,-)	
PPV-2	RS	16/30	32.8	16.1, 35.8	22/30	34.3	28.2, 35.8	8/30	32.2	28.1, 35.7	46/90	33.4	16.1, 35.8
		(53%)	(30.6, 34.0)		(73%)	(32.3, 35.0)		(27%)	(30.2, 34.2)		(51%)	(31.2, 34.6)	
	TTF	30/30	32.7	16.6, 35.9	29/30	29.6	19.6, 33.6	30/30	28.6	19.7, 32.6	89/90	29.6	16.6, 35.9
		(100%)	(29.8, 33.5)		(97%)	(28.2, 30.7)		(100%)	(26.1, 30.0)		(99%)	(28.1, 31.7)	
IAV	ONS	0/30	-	-	30/30	21.9	17.0, 31.9	3/30	28.9	28.5, 35.0	33/90	22.2	17.0, 35.0
		(0%)	-		(100%)	(20.1, 24.4)		(10%)	(28.7, 31.9)		(37%)	(20.2, 24.6)	
	TTF	0/30	-	-	30/30	26.7	18.1, 31.4	4/30	34.4	32.4, 35.9	34/90	26.9	18.1, 35.9
		(0%)	-		(100%)	(23.9, 29.0)		(13%)	(33.3, 35.3)		(38%)	(24.1, 30.1)	
*L. intracellularis*	RS	0/30	-	-	0/30	-	-	0/30	-	-	0/90	-	-
		(0%)	-		(0%)	-		(0%)	-		(0%)	-	
	TTF	0/30	-	-	0/30	-	-	5/30	35.5	34.3, 35.5	5/90	35.5	34.3, 35.5
		(0%)	-		(0%)	-		(17%)	(35.3, 35.5)		(6%)	(35.3, 35.5)	

* TTF = tongue tip fluid; ONS = oronasal swab; RS = rectal swab; SILN = superficial inguinal lymph node; ICS = intracardiac serum. ¶ ICS from three pigs did not yield enough quantity to obtain a result.

**Table 2:** Estimated PRRSV-2 sensitivity (Se), specificity (Sp), positive predictive value (Ppv) and negative predictive value (Npv) of three specimens compared to intracardiac serum by visit.

Specimen	Measure	Farm and Visits (95% CI)			Overall (95% CI)
		Farm 1		Farm 2	
		Visit 1	Visit 2	Visit 3	
Tongue tip fluid	Se	100% (88%, 100%)	84% (60%, 97%)	100% (87%, 100%)	96% (88%, 99%)
	Sp	NA	9% (0%, 41%)	0% (0%, 71%)	7% (0.1%, 34%)
	Ppv	100% (88%, 100%)	62% (41%, 80%)	90% (73%, 98%)	84% (75%, 91%)
	Npv	NA	25% (1%,81%)	NA	25% (0.6%, 81%)
Oronasal swab	Se	100% (88%, 100%)	74% (49%, 91%)	100% (87%, 100%)	93% (85%, 98%)
	Sp	NA	73% (39%, 94%)	0% (0%, 71%)	57% (29%, 82%)
	Ppv	100% (88%, 100%)	82% (56%, 96%)	90% (73%, 98%)	92% (83%, 97%)
	Npv	NA	62% (32%, 86%)	NA	62% (32%, 86%)
Superficial inguinal lymph node	Se	100% (88%, 100%)	100% (82%, 100%)	100% (87%, 100%)	100% (95%, 100%)
	Sp	NA	9% (2%, 41%)	0% (0%, 71%)	7% (0.1%, 34%)
	Ppv	100% (88%, 100%)	66% (46%, 82%)	90% (73%, 98%)	85% (76%, 92%)
	Npv	NA	100% (3%, 100%)	NA	100% (2.5%, 100%)

NA = not applicable.

**Table 3:** Estimated agreement, Cohen’s kappa, and global agreement between specimens’ pathogen detection.

Pathogen	Specimens *	Cohen’s Kappa (95% CI)	COHEN’S Kappa *p*-Value	Global Agreement
PRRSV-2	ICS and TTF	0.04 (−0.16, 0.24)	0.67	82%
	ICS and ONS	0.52 (0.27, 0.77)	<0.001	87%
	ICS and SILN	0.11 (−0.09, 0.32)	0.27	85%
	ONS and TTF	0.31 (0.02, 0.59)	0.04	87%
	ONS and SILN	0.12 (−0.09, 0.35)	0.27	87%
	SILN and TTF	−0.02 (−0.05, 0.01)	0.22	94%
PCV-2	SILN and TTF	0.2 (0.04, 0.36)	0.01	66%
PCV-3	SILN and TTF	NA	NA	100%
PPV-1	RS and TTF	NA	NA	99%
PPV-2	RS and TTF	−0.02 (−0.07, 0.02)	0.31	50%
IAV	ONS and TTF	0.98 (0.93, 1.00)	0.00	99%
*L. intracellularis*	RS and TTF	0.00 (0.00, 0.00)	NA	94%

* TTF = tongue tip fluid; ONS = oronasal swab; RS = rectal swab; SILN = superficial inguinal lymph node; ICS = intracardiac serum.

## Data Availability

Data used to calculate sensitivity, specificity, positive and negative predictive values, and proportions are included in this published article. Additional data that support the findings of this study are available from the corresponding author upon reasonable request.

## Abbreviations

The following abbreviations are used in this manuscript:

TT	Tongue tip
TTF	Tongue tip fluid
ONS	Oronasal swab
SILN	Superficial inguinal lymph node
RS	Rectal swab
ICB	Intracardiac blood
ICS	Intracardiac serum
PRRS	Porcine reproductive and respiratory syndrome
PCV	Porcine circovirus
PPV	Porcine parvovirus
IAV	Influenza A virus
RT	Reverse transcription
PCR	Polymerase chain reaction
AASV	American Association of Swine Veterinarians
WOA	Weeks of age
AVMA	American Veterinary Medical Association
UMN	University of Minnesota
VDL	Veterinary diagnostic laboratory
Se	Sensitivity
Sp	Specificity
Ppv	Positive predictive value
Npv	Negative predictive value
CI	Confidence interval
